# Evaluating the implementation and impact of navigator-supported remote symptom monitoring and management: a protocol for a hybrid type 2 clinical trial

**DOI:** 10.1186/s12913-022-07914-6

**Published:** 2022-04-22

**Authors:** Gabrielle B. Rocque, J. Nicholas Dionne-Odom, Angela M. Stover, Casey L. Daniel, Andres Azuero, Chao-Hui Sylvia Huang, Stacey A. Ingram, Jeffrey A. Franks, Nicole E. Caston, D’ Ambra N. Dent, Ethan M. Basch, Bradford E. Jackson, Doris Howell, Bryan J. Weiner, Jennifer Young Pierce

**Affiliations:** 1 Department of Medicine, Division of Hematology and Oncology, University of Alabama at Birmingham, 1824 6th Avenue South, 35924-3300 – WTI 240E, Birmingham, AL USA; 2 Department of Medicine, Division of Gerontology, Geriatrics, and Palliative Care, University of Alabama at Birmingham, Birmingham, AL USA; 3 O’Neal Comprehensive Cancer Center, Birmingham, AL USA; 4 University of Alabama at Birmingham School of Nursing, Birmingham, AL USA; 5 University of South Alabama Mitchell Cancer Institute, Mobile, AL USA; 6 Supportive Care, Princess Margaret Cancer Centre Research Institute, Toronto, Ontario Canada; 7 Lineberger Comprehensive Cancer Center, University of North Carolina at Chapel Hill, NC Chapel Hill, USA; 8 Department of Health Systems and Population Health, University of Washington, Seattle, Washington, USA

**Keywords:** Remote symptom monitoring, Payment reform, Patient-reported outcomes, Real-world data, Implementation strategies

## Abstract

**Background:**

Symptoms in patients with advanced cancer are often inadequately captured during encounters with the healthcare team. Emerging evidence demonstrates that weekly electronic home-based patient-reported symptom monitoring with automated alerts to clinicians reduces healthcare utilization, improves health-related quality of life, and lengthens survival. However, oncology practices have lagged in adopting remote symptom monitoring into routine practice, where specific patient populations may have unique barriers. One approach to overcoming barriers is utilizing resources from value-based payment models, such as patient navigators who are ideally positioned to assume a leadership role in remote symptom monitoring implementation. This implementation approach has not been tested in standard of care, and thus optimal implementation strategies are needed for large-scale roll-out.

**Methods:**

This hybrid type 2 study design evaluates the implementation and effectiveness of remote symptom monitoring for all patients and for diverse populations in two Southern academic medical centers from 2021 to 2026. This study will utilize a pragmatic approach, evaluating real-world data collected during routine care for quantitative implementation and patient outcomes. The Consolidated Framework for Implementation Research (CFIR) will be used to conduct a qualitative evaluation at key time points to assess barriers and facilitators, implementation strategies, fidelity to implementation strategies, and perceived utility of these strategies. We will use a mixed-methods approach for data interpretation to finalize a formal implementation blueprint.

**Discussion:**

This pragmatic evaluation of real-world implementation of remote symptom monitoring will generate a blueprint for future efforts to scale interventions across health systems with diverse patient populations within value-based healthcare models.

**Trial registration:**

NCT04809740; date of registration 3/22/2021.

**Supplementary Information:**

The online version contains supplementary material available at 10.1186/s12913-022-07914-6.

## Background

Patients with cancer experience a myriad of symptoms, many of which are inadequately assessed during encounters with the healthcare team. Previous studies showed that patients with cancer report symptom severity up to 40% higher than their physician’s report and a substantial proportion of symptoms are missed entirely [1–3]. Patient-reported outcomes (PROs) are emerging as a potential solution to address this gap in symptom identification and ultimately to improve patient management. PROs are defined as “information about a patient’s health status that comes directly from the patient,” which can include symptoms as well as other key patient-reported data such as quality of life and physical function [4]. In patients with advanced cancer, Basch and colleagues assessed weekly electronic home-based PRO symptom monitoring with automated alerts to clinicians (remote symptom monitoring) within a single-site randomized clinical trial, finding reduced emergency department (ED) and hospital visits, improved health-related quality of life, and a 5-month increase in overall survival [5]. Other studies of ePROs have similarly found benefits in terms of efficiency of symptom assessment [6–9]; patient-clinician communication and satisfaction [10–12]; symptom control and well-being [9, 13–18]; frequency of hospitalizations [19]; and survival [20].

Despite evidence of benefit, general oncology practices have lagged in adopting remote symptom monitoring into routine practice [21]. Prior studies that utilized research funding to support remote symptom monitoring administration, achieving high compliance with 80–85% of patients in studies being willing and able to self-report symptoms in remote symptom monitoring trials [16]. However, patients enrolling in randomized trials using remote symptom monitoring may not represent the general cancer population and/or barriers to participation exist for patients who are not on clinical trials. For example, only 9% of one study’s participants were Black, and few lived in rural areas [5]. Limited data are available on the use of remote symptom monitoring in diverse populations and incorporating remote symptom monitoring into routine clinical care, at scale, is expected to require considerable staff engagement and modifications to the healthcare delivery system given the complex nature of the intervention. Thus, a substantial knowledge gap remains regarding optimal strategies for remote symptom monitoring implementation in real-world settings where all patients in a cancer center participate, including diverse populations that may differ in their participation, barriers, and outcomes.

One emergent trend that could potentially support the implementation of remote symptom monitoring in real-world settings is the transition toward value-based health care. In 2016, the Centers for Medicare and Medicaid Innovation launched the Oncology Care Model (OCM), a nationwide payment reform demonstration project [22]. The OCM and the proposed Oncology Care First Model (Medicare’s proposed payment reform project) require use of navigators for cancer care coordination in all > 100 participating practices across the US [22]. The patient navigation workforce is ideally positioned to assume a leadership role in remote symptom monitoring implementation, as collecting and responding to PROs aligns with their designated roles and responsibilities, such as assessing functional status, depression, and distress while patients are present in clinic [23]. The goals of navigation include enhancing care coordination and proactively managing patient concerns [24]. Patient navigation programs have proven efficacious for increasing access to care [25, 26], care coordination [27], symptom management [28, 29], and reducing cost [30, 31]. These demonstrated benefits contribute to the growing number of nurse and lay (non-clinical) navigation programs, particularly for practices in communities with limited resources [32].

**Fig. 1:**
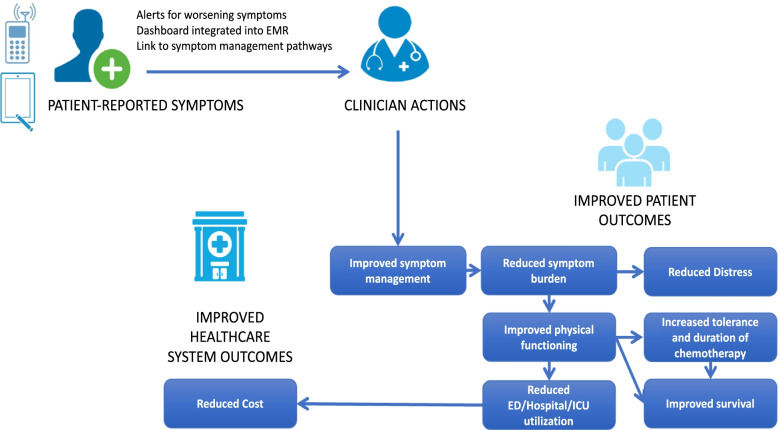
Conceptual model for improvement in outcomes from use of remote symptom monitoring

The OCM recommendation to use navigators in cancer centers was based, in part, on a 2012 Center for Medicare and Medicaid Innovation project conducted by the University of Alabama at Birmingham (UAB) and University of South Alabama Mitchell Cancer Institute (MCI), which showed lower cost with higher quality metrics from implementation of patient navigation services across the southeastern U.S. [24, 33] We developed a lay-navigator led approach to remote symptom monitoring which is being implemented as part of routine care at UAB and MCI with support from payment reform initiatives and grant funding. Remote symptom monitoring is predicated on hypotheses that electronically captured patient-reported symptoms will result in increased clinical team awareness and prompt clinical action, resulting in improved symptom management and reduced symptom burden. These improvements are expected to have the downstream effects of lowering distress and improving physical function, which will translate to improved tolerance of chemotherapy, reduced hospital utilization and cost, and improved survival (Fig. 1). This pragmatic study will evaluate implementation of navigator-delivered remote symptom monitoring for all patients with cancer across two practice sites (Aim 1); examine the barriers, facilitators, and implementation strategies used in implementing navigator-delivered remote symptom monitoring (Aim 2); and assess the impact of remote symptom monitoring on clinical and utilization outcomes for a general cancer population receiving medical therapies (e.g., chemotherapy, immunotherapy, targeted therapy) (Aim 3) for all patients and for diverse populations. This study will ultimately generate a blueprint of implementation strategies for sustainable, navigator-delivered remote symptom monitoring that does not rely on research funding.

## Methods

### Study design

**Fig. 2:**
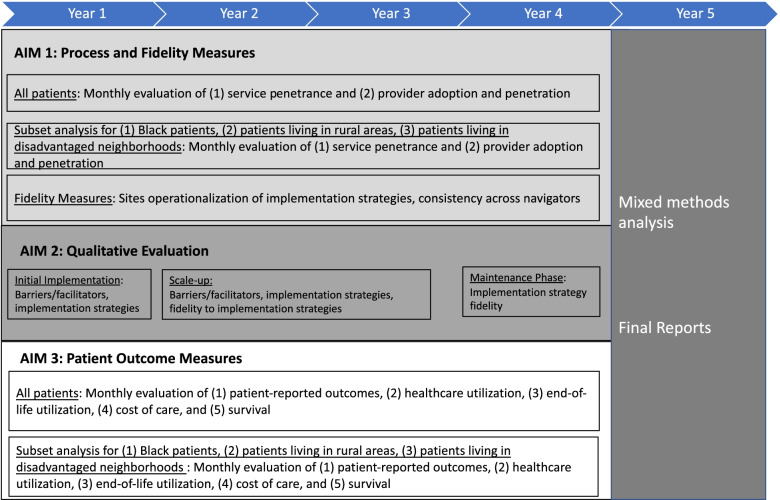
Study schema

This hybrid type 2 study design [34], simultaneously evaluates the implementation and the effectiveness of remote symptom monitoring as standard of care. Recruitment to the system-wide phase of implementation began in May 2021. At the time of manuscript submission, the remote symptom monitoring implementation and evaluation of the program is actively ongoing and will continue through May 2026. This mixed methods study will have two key components: (1) secondary data analysis to evaluate key implementation (e.g., service penetrance and provider adoption/penetrance) and patient outcomes (e.g., symptom burden, healthcare utilization, end-of-life care, cost of care, survival); and (2) qualitative evaluation at key time points will be utilized to assess barriers and facilitators to remote symptom monitoring, implementation strategies, fidelity to implementation strategies, and perceived utility of these strategies (Table 1). For quantitative outcomes, this study will utilize a pragmatic approach evaluating real-world data collected as part of routine care. We will use a mixed methods approach (QUAL + QUAN) [35] to triangulate the qualitative and quantitative findings using parallel side-by-side comparisons to integrate both data sources to interpret findings and to finalize a formal implementation blueprint. The study schema is shown in Fig. 2**.**

**Table 1:** Outcomes for Aims 1,2,3

Aim	Outcome Type	Concept	Unit of assessment	Evaluation Metrics	Data Source	Timing
1	Implementation Outcomes	Intervention fidelity	Sites	Review of use of intervention details	Implementation team	Monthly
			Clinicians	Training completion, consistency of ePROs completed fully, and response to alerts	EMR and ePRO data	Annually
		Service penetration	Practice	% of eligible patients enrolled; % of patients completing ≥1 ePRO report; % of expected ePRO reports completed per patient; % of patients with reports at 3 and 6 months	EMR and ePRO data	Monthly
		Provider adoption/ penetration	Clinicians	% of clinical teams participating in training; % responding to alerts; time to response, type of response to alerts	Training logs, EMR nursing documentation, surveys	Monthly
2	Qualitative Outcomes	Barriers and facilitators of remote symptom monitoring	Patients and Clinicians	Participant perceptions of remote symptom monitoring implementation	Patient and staff interviews	Annually
		Implementation Strategy Fidelity	Clinicians	How participants operationalized implementation strategies, why they used this approach, how did they adapt strategies	Patient and staff interviews	Annually
		Implementation Strategy Benefit	Clinicians	Participant perception of how well strategies work in addressing implementation barriers	Patient and staff interviews	Annually
3	Patient-Reported Outcomes	Symptom burden^a^	Patients	% of patients who trigger alert, 6-month symptom trajectory; types of alerts	ePRO data from clinical encounters	3 and 6 months after initiating treatment
		Patient functioning		Association between intervention participation and Eastern Cooperative Oncology Group performance status		
		Distress, Depression		Association between intervention and distress and depression scores		
	Healthcare Utilization	ED visits	Patients	Proportion with ED visit	Claims data	3 and 6 months after initiating treatment
		Hospitalizations		Proportion hospitalized		
		ICU admissions		Proportion with ICU admission		
	End-of-Life Care	ED visits	Deceased patients	Proportion with ED visit	Claims data	Last 30 days of life
		Hospitalizations		Proportion hospitalized		
		ICU admissions		Proportion with ICU admission		
		Cost		Total cost of care in last 30 days of life		
	Cost of Care	Total cost of healthcare	Patients	Total cost of care to payer and patient cost responsibility at 3 months and 6 months post-treatment initiation	Claims data	3 and 6 months after initiating treatment
	Survival	Survival	Patients	Overall survival	Claims data	End of study

^a^10 common symptoms, available for patients receiving remote symptom monitoring electronic patient reported outcome (ePRO) surveys. *EMR* Electronic medical record, *ED* Emergency department, *ICU* Intensive care unit

This study was approved by a central Institutional Review Board from UAB, which served as the IRB record for this project with secondary approval from MCI and University of North Carolina Chapel Hill. Evaluating the implementation and impact of navigator-delivered remote symptom monitoring trial is supported by the National Institute for Nursing Research (1R01NR019058–01).

### Implementation setting

UAB and MCI are the academic medical institutions in the state of Alabama and treat a largely nonoverlapping but similar population. These institutions serve historically diverse populations, including Black patients (25%), rural residents (30%), socioeconomically disadvantaged people (10% dual eligible for Medicare and Medicaid), and 15% of residents have a high school education or less [36]. Alabama has a large number of census block groups with high Area Deprivation Index scores, reflecting the low socioeconomic status of the state [37]. Internet connectivity has been a challenge in Alabama, but in May 2019, Alabama passed House Bill 400 which allows electrical providers to use existing and future power networks for high-speed internet in rural communities [38]. In recent years, more than $62 M in grants were awarded to community partners for the provision of high-speed internet in Alabama communities with limited access [39]. For our study in routine care, philanthropic support is available from Blue Cross Blue Shield of Alabama (BCBS-AL) for mobile phones for patients without high-speed internet access at home.

### Standard-of-care components

#### Remote symptom monitoring implementation

**Fig. 3:**
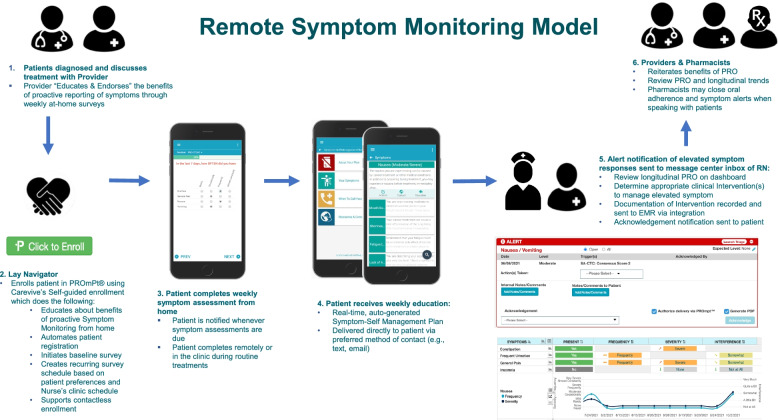
Remote symptom monitoring process

Remote symptom monitoring will be implemented as standard of care with all patients offered the intervention, thus no consent will be required. The remote symptom monitoring process and technology components are shown in Fig. 3. Physicians will introduce the program to new patients as an approach for routine monitoring. Patients who are on chemotherapy, targeted therapy, or immunotherapy will be approached for enrollment by lay navigators. The navigator will explain the rationale for remote symptom monitoring, help the patient select the e-mail or text option for symptom surveys, and initiate the video-based self-enrollment process. The navigators guide the patients through the technical aspects of participation. Patients will be allowed to opt out of participation. After enrollment, patients will be asked to complete a weekly symptom assessment using the ePRO version of the Common Terminology Criteria for Adverse Events (PRO-CTCAE) [40]. If patients do not complete their survey, two reminders will be sent, followed by a notification to the lay navigator to call the patient. Lay navigators will be responsible for retention in remote symptom monitoring, which will include monitoring for survey non-completion and assessing reasons for lack of participation. If severe symptoms or a change in symptoms are reported, an automatic notification will be sent to the nurse or nurse navigator as a message in the electronic medical record (EMR). The nurse will call the patient, coordinate care, and communicate with the physician as needed. In the clinic, members of the clinical team will review symptoms within an integrated dashboard in the EMR and adjust management as needed.

#### Staff training

We will offer multiple provider remote symptom monitoring training sessions and keep detailed logs of the number of providers participating in training. The initial training will be led by the PI or site PI and will include a 30-min didactic presentation on the purpose of remote symptom monitoring, the planned workflow, preliminary data from participating institutions, and a brief overview of the project evaluation plan. If a provider does not attend training, we will document the reason for declining participation. The frequency and percentage of clinical teams (physicians, nurses, navigators) participating in training will be recorded. Of note, > 90% participation from staff is expected because training will be integrated into routine staff education. These didactic trainings will be hands-on, practical training led by a super user of the platform. For lay navigators, this includes shadowing followed by observed navigator-led program enrollment over the course of 2–3 weeks. For nurses, hands-on training is completed in approximately 15–20 min and focused on locating alerts in the medical record, closing alerts, and reviewing summary dashboards. Additionally, the super user will also complete routine check-ins with the clinical teams and provide additional review of technology components as needed.

### Research components on implementation

#### Implementation frameworks

The Consolidated Framework for Implementation Research (CFIR) by Damschroder and colleagues is ideally positioned to frame evaluation of multi-level barriers to implementation and implementation strategies deployed as part of remote symptom monitoring [41]. The CFIR starts with the key intervention characteristics, but also hypothesizes that the inner setting, outer setting, characteristics of individuals involved, and implementation process all influence successful implementation [41]. The CFIR will also guide qualitative evaluations. Proctor’s Implementation Outcome Framework [42] will be used in assessment of implementation outcomes, providing a guide to practical implementation outcomes for both ongoing quality monitoring as well as to inform future practice on expected implementation. We will operationalize Proctor’s outcomes using Stover’s PRO Implementation science metrics for evaluating PRO initiatives in usual care settings. These metrics integrate prior frameworks to assess the relationship between remote symptom monitoring implementation strategies, proximal variables or mediators, implementation science outcomes, and patient clinical outcomes [43].

#### Implementation strategy selection

We anticipate barriers (e.g., potential for staff burden) to be encountered during implementation, resulting in the need for additional implementation strategies. We will use the CFIR-ERIC (Expert Recommendations for Implementing Change) Barrier Busting Query Tool V 1.0 and Intervention Mapping [44] to identify appropriate implementation strategies to overcome additional barriers encountered [45]. This approach has been successfully used by Howell and colleagues in implementation of self-management support for symptoms in patients with cancer [46]. Intervention mapping is a rigorous strategy design approach combining theory, evidence, and practice characteristics, which seeks to identify any additional implementation strategies necessary for targeting barriers identified within the proposed study’s qualitative interviews.

### Implementation outcome evaluation (quantitative)

#### Evaluate implementation of navigator-delivered remote symptom monitoring for all cancer patients across multiple practice sites (aim 1)

##### Sampling and recruitment

This study will include two groups of participants identified through medical record review: (1) patients and (2) clinical team members. The patient group will be composed of all adults receiving their initial treatment with chemotherapy, targeted therapies, or immunotherapies at UAB or MCI from 2021 to 2026 (regardless of whether they completed remote symptom monitoring). Patients can receive concurrent treatments (e.g. radiation, surgery). Patients aged 18 years or older, all races and ethnicities, and all insurance types will be included for the duration of their treatment. Patients who are seen for a second opinion or to receive hormone therapy alone will be excluded. For clinical teams, we will include all medical and gynecologic oncologists, nurses, and navigators (nurse and lay) who care for patients receiving chemotherapy. There are no exclusion criteria for providers.

### Data collection

Data from this study will be collected at multiple levels, including the cancer center, clinical team, and patients. Training materials, logs, and minutes will be captured and reviewed on an ongoing basis. The following data from the EMR will be abstracted: age, sex, race and ethnicity, insurance status, home address, cancer type, cancer stage, diagnosis date, treatments received, and dates of treatments. Home address will be utilized to identify urban or rural residence using Rural-Urban Commuting Area (RUCA) codes and to characterize their Area Deprivation Index scores, which is a combined measure of socioeconomic disadvantage [47]. The providers’ roles will be identified and recorded using personnel lists available through clinic supervisors (e.g. physician, clinic nurse, nurse navigator, lay navigator). Remote symptom monitoring data will be abstracted from the PROmpt™ system (Patient Reported Outcome mobile platform technology), which is integrated into the EMR. From this system, data will be abstracted monthly and will include: member of the team responsible for survey compliance, individual responsible for alert management, physician, date enrolled, reason for not enrolling, survey administration date, date of reminders for survey completion, survey completion date, survey responses, alerts, time to close alert, and responses to alert. Additional chart review will be completed as needed to supplement missing data.

### Outcomes

#### System changes

Given the multi-level influences on our implementation outcomes, we will record in real-time the date and details of any changes in team staffing or organization, institutional policy changes, or national policy changes. Staffing and institutional policy changes may be provided by a member of the service line or OCM leadership teams.

#### Implementation outcomes

Implementation outcomes are based on Proctor’s Implementation Framework [42], with personalization of these outcomes using Stover’s PRO implementation science outcome metrics [43]. Patient service penetrance will be evaluated using the following outcomes: (1) percentage of patients approached and enrolled of all new adult patients initiating treatment; (2) percentage completing at least one remote symptom monitoring assessment; (3) percentage completing assessments at 3 months, 6 months, and percentage of all expected surveys. A 75% completion rate is expected, which is a 10% reduction observed in randomized trials due to application of ePRO surveys in a real-world setting [16]. Provider adoption and penetration will be assessed using training logs and medical record documentation. We will include the following outcomes: (1) percentage of alerts with a response (with > 90% response rates to remote symptom monitoring symptom alerts expected because remote symptom monitoring will be integrated into the clinical team’s workflow and EMR); (2) response time for alerts; (3) types of responses to alerts. Reports on selected outcomes will be provided monthly to the implementation team as an audit and feedback mechanism to allow for identification of implementation challenges.

#### Intervention fidelity

Recognizing minor adaptations will be necessary to move implementation from a clinical trial to real-world clinics, intervention fidelity will be tracked through monthly reviews. Adaptations will be reviewed with the research team at least quarterly for the purpose of clearly reporting adaptations to evidence-based interventions using the FRAME (Framework for Modifications and Adaptions) by Stirman and colleagues [48]. We will include details about adaptations to the intervention within a final implementation blueprint.

#### Analysis

We will first descriptively summarize baseline health system characteristics, participant demographics, and study outcomes. We will examine differences in patient characteristics between those who participate and those who do not using bivariate measures of association (e.g., Cohen’s d, Cramer’s V). For patient outcomes, site and navigation team will be treated as fixed effects when needed, as all navigation teams will be included. The primary analysis will be conducted using logistic regression models to estimate the service penetration proportions of interest throughout the project. Model-predicted means and inverse-link transformations will be used to estimate the proportions of interest and respective 95% confidence intervals. Secondary analysis for patient outcomes will be conducted using logistic regression models to evaluate the association between patient characteristics and penetration outcomes. Patient characteristics will include age, sex, race and ethnicity, rurality (estimated using RUCA codes), driving distance from cancer care site, and socio-economic disadvantage status (estimated using the Area Deprivation Index). For clinician metrics, generalized linear mixed models with random effect for clinician team will be used to estimate the monthly response to alerts and time to response. A False Discovery Rate approach [49] will be used to correct for multiple inference when appropriate (10% FDR).

#### Sample size considerations

UAB and MCI see approximately 4000 patients and 2600 new patients per year, respectively. We anticipate approximately 35% of patients to be receiving chemotherapy, thus > 2000 patients will be eligible to participate in remote symptom monitoring each year. We expect implementation will increase over the 5 year study period. In Year 1, we anticipate at least 30% of patients to be approached (*n* = 600) with increases by 10% each year to 70% (*n* = 1400) at Year 5, for a total of 5000 patients over the duration of the funding period. As an opt-out program, we predict that 75% of patients approached will be willing to complete ePROs based on our prior PRO work and recent pilot. Thus, we anticipate at least 3750 will enroll in remote symptom monitoring. Under these assumptions, the expected large sample size provides high power and precision; however, inferences apply to patient populations from similar systems and in adjacent geographical areas. For the expected 40% increase in patients approached between Year 1 and Year 5, the 95% confidence interval is 36–44% (from 30 to 70%). For the expected 75% patient participation if approached in Years 1–5, the 95% confidence interval is 73–77%.

### Implementation outcome evaluation (qualitative)

#### Examine the barriers, facilitators, and implementation strategies used in implementing navigator-delivered remote symptom monitoring (aim 2)

##### Sampling and recruitment

For patients, each health system will identify up to 20 patients in Years 1 (baseline), 2 (early implementation), and 4 (maintenance) to participate in individual interviews. Patient participants will be selected using the purposive sampling technique [50] for variation in perspectives. We expect engagement of patients of different races and ethnicities, distances traveled for cancer care, levels of prior computer use, education levels, and insurance types. For providers, sites will identify a convenience sample of up to ten staff for interviews at each site. The interviewed staff will include the nursing supervisor and champions representing physicians, nurses, lay navigators, nurse navigators, and administrators. Different patients and providers will be selected at each time point. The sample will be expanded in Year 4 if thematic saturation is not reached for the key themes of barriers, implementation strategies, and benefit of strategies to address barriers [51].

##### Data collection and outcomes

A multi-disciplinary team including experts in medical oncology, gynecologic oncology, psychology, social and behavioral science, and implementation science will generate an a priori thematic schema. Interviews will be designed to elucidate (1) acceptability and (2) barriers to remote symptom monitoring implementation using the determinants; (3) approaches to operationalizing implementation strategies using existing literature on implementation strategies and ePRO delivery within clinical trials; and (4) perceptions of how well selected implementation strategies addressed barriers and recommendations for improvement. In-depth interviews will be led jointly by the site PIs and experts in program implementation using interview guides developed by the multidisciplinary research team. The interviews will elucidate important contextual factors based on the CFIR, barriers to implementation, and how implementation strategies are used to overcome these barriers, which may influence health systems’ ability to optimally implement and sustain remote symptom monitoring and ultimately improve outcomes. Examples of implementation strategies and associated CFIR targets, barriers, team member responsible, and actions are shown in Table 2. All audio files will be uploaded to a study computer and stored in password-protected files. Audio files will be transcribed verbatim.

**Table 2:** Examples of planned implementation strategies

CFIR Targets	Barrier	Implementation Strategy	Team member(s) responsible	Action	Qualitative Prompts for Aim 2
Inner setting, individuals involved	Physician champions are not aware of implementation plan	Identify and prepare champions	Research team, Carevive	Educate staff on rationale and details of the intervention	“What have you and your team done to build ‘buy in’ for your site’s remote symptom monitoring project?”
Process of implementation	Technical issues may arise during implementation	Centralize technical assistance	Research team, Carevive	Provide technical support and conduct weekly team meetings to address challenges to implementation	“What has your team done to provide support and educate navigators and others to troubleshoot technical issues during implementation?”
Intervention characteristics, individuals involved	Remote symptom monitoring are not utilized by navigators	Revise professional roles	Navigators	Assign responsibility for patient enrollment, monitoring, and responding to ePROs to navigator teams	“How did the additional role of remote symptom monitoring implementation impact the work of the navigators?”
Intervention characteristics, individuals involved	Patients are unfamiliar with the technology	Change service site	Navigators	Assign patients to complete symptom ePROs at home	“How did changing the location of symptom collection from clinic only to home and clinic impact symptom management?”
Intervention characteristics	ePRO data is unavailable for clinicians	Facilitate display of clinical data to providers	Carevive, Cerner	Use dashboard within EMR	“What was helpful within dashboard?”
Process of implementation	The amount of data may be overwhelming for clinicians	Develop and organize quality monitoring and improvement	Research team, Carevive	Create reports to be used in weekly meetings	“How has your team responded to quality monitoring reports?”
Inner setting, process of implementation	Patients may stop completing surveys; providers may stop alert response	Audit and feedback	Research team, Carevive	Create reports	“How did the feedback reports (or alerts) impact your ability to manage patient symptoms and implement remote symptom monitoring?”
Process of implementation	There may be unexpected implementation challenges	Use implementation advisors	Dr. Stover, Dr. Howell, Dr. Weiner, research team	Calls with Dr. Stover	“How did the implementation advisor help the implementation?”
All settings	Sites may have different applications of implementation strategies	Create a learning collaborative	Research team	Bi-monthly calls between implementation teams at UAB and MCI	“What were the benefits and challenges of participating in calls with your partner health system?”
Process of implementation	There is an ongoing need for continuous system improvement	Plan for sustainability	Research team, Carevive, Blue Cross Blue Shield, administrators	Stakeholder engagement, technology improvements	“What were the key components you needed to be able to continue using remote symptom monitoring?”

Implementation team includes the Principal Investigator, co-investigators, Oncology Care Model administrative director, navigator supervisors, and nurse supervisors. *ePRO* Electronic patient-reported outcomes; *EMR* Electronic medical record, *UAB* University of Alabama at Birmingham, *MCI* Mitchell Cancer Institute

Changes to the base implementation strategy package will be considered an adaptation, which will be recorded using the FRAME framework by Stirman and colleagues for reporting adaptations to evidence-based interventions [48]. Implementation strategy fidelity will be assessed through formal tracking of site base implementation strategies, added implementation strategies, adaptations made and why, and perceptions of the utility of strategies deployed included in the implementation blueprint.

##### Analysis

The analytic strategy is primarily informed by content analysis, which examines the language to classify the text into categories that represent key concepts within the interviews [52]. Qualitative coding and content analysis will consist of identifying quotations which express themes related to remote symptom monitoring barriers, use of implementation strategies, fidelity to implementation strategies, and perception of specific implementation strategy benefits [53]. In the initial stages of coding, two independent coders with health behavior and medical anthropology expertise will read the transcripts and develop an open coding scheme, which is the process of labeling portions of text to identify all ideas, themes, and issues suggested by the data [54]. Analytic codes constructed in the context of open coding are provisional and will be grounded within the data [55]. The final version of the coding schema will be reviewed and finalized by the multi-disciplinary team, which will include the two primary coders, an oncologist, a gynecologic oncologist, a nurse researcher, and implementation scientists. The two primary coders will subsequently use NVivo software (QRS International) to conduct “focused coding,” which includes a detailed analysis of themes identified during open coding. Any discrepancies will be resolved by a third coder. The process will be repeated until thematic saturation is reached, where no new categories or relevant themes emerge [51]. Data from interviews will be analyzed at an aggregate and health system-specific level. Summaries will be reviewed with the multi-disciplinary team and used to modify the implementation blueprint by adding or removing implementation strategies.

##### Sample size considerations

Sampling for qualitative inquires is sequential and targeted to individuals who can provide insights on the study processes. Typically, qualitative approaches involve < 50 participants [56]. If thematic saturation is not reached, we will increase the number of participants. Access to a diverse group of stakeholders in the two health systems will provide a sufficient participant pool to carry out the inquiry. If differences in qualitative or quantitative outcomes are observed in Years 1–3 for a specific sub-population (e.g. older adults, patients with low socioeconomic status), we will expand Years 4–5 qualitative interviews to include an additional 10–20 participants of these sub-populations.

### Patient outcome evaluation (quantitative)

#### Assess the impact of remote symptom monitoring on clinical and utilization outcomes (aim 3)

##### Sampling and recruitment

Patients included in Aim 1 will be included in Aim 3.

##### Data collection

In addition to data described above, Aim 3 data will include claims data provided quarterly by Medicare and every 6 months from Blue Cross Blue Shield of Alabama. These data will be utilized to assess patient characteristics, clinical characteristics, healthcare utilization, survival, and cost to the payer.

##### Patient outcomes

Outcomes will include patient-reported functioning and distress (patient surveys); rates of healthcare utilization (emergency department visits, hospitalizations, intensive care unit admissions), treatment duration, and total cost to payer (claims data); and overall survival (claims data).

##### Analysis

Patient-reported outcomes and utilization trends will be described for all patients, including patients receiving and not receiving remote symptom monitoring. Latent class models stratified by cancer type will be used to explore 6-month symptom trajectory groups and the relationships between group membership and relevant covariates such as age, cancer type/stage, and socioeconomic disadvantage status. We will estimate a propensity score (the probability of being a remote symptom monitoring participant given the values of relevant patient characteristics) and use it to match remote symptom monitoring participants with historical controls using radius matching. If needed, we will use matching with replacement to include as many remote symptom monitoring patients as possible in the analyses. To minimize the potential confounding effect of change or improvement of cancer therapy over time, we will restrict the pool of controls to patients initiating treatment up to 3 years before the implementation of remote symptom monitoring intervention. Given this time restriction, it is possible that matching with replacement (i.e., a control patient could be matched with more than one ePRO patient) will be needed to include as many remote symptom monitoring patients as possible. We will then use generalized linear or generalized linear mixed models, as appropriate, to conduct between-group comparisons on ePROs, healthcare utilization, and cost of care. For survival analysis, we will recode and censor the survival time of the controls as appropriate to match the potential follow-up time of the remote symptom monitoring patients. We will use time-to-event to estimate and provide inferences on survival differences. A False Discovery Rate approach will be used to correct for multiple inference when appropriate (10% FDR).

### Synthesis into implementation blueprint

An initial set of implementation strategies to be used by sites was identified for the initial “formal implementation blueprint.” [57, 58] Table 2 includes CFIR targets, planned implementation strategies to address barriers, and qualitative evaluation prompts. The selected strategies focus on building buy-in, educating both stakeholders and participants in the intervention, restructuring the workforce and technology to facilitate uptake, and developing quality management strategies to facilitate successful implementation [57, 58]. To promote intervention sustainability, the investigative team will invite key external stakeholders to participate in annual meetings to discuss implementation progress, review implementation strategies, and assess available data on patient and health system outcomes. Stakeholders will include participants from the investigative team, electronic health record company, patient-reported outcome platform company, BCBS-AL, UAB and MCI cancer center directors, key administrative leadership, the director for the Office of Community Outreach, and a patient advocate. Detailed meeting notes will be captured. These discussions will generate continued engagement and support for the intervention and improvements to the implementation blueprint. We will provide a final implementation blueprint to stakeholders that can be used with other health systems for implementing navigator-led remote symptom monitoring.

## Discussion

Both Medicare’s proposed Oncology First Model and the American Society of Clinical Oncology’s Oncology Medical Home demonstration project [59, 60] are proposed to require implementation of patient-reported outcomes in routine care. This policy context provides a tremendous opportunity in terms of both resources and cultural pressure for implementation. At the same time, inclusion of remote symptom monitoring in payment models exposes vulnerabilities due to the lack of rigorous data on implementation outcomes available for oncology practices. Without data to guide implementation, timely integration of this intervention into practice will be challenging. The application of implementation science methodology to practice transformation activities required within payment reform fills a substantial knowledge gap. In addition, the unique alignment of financial incentives for payers, practices, and technology companies creates an opportunity to educate these stakeholders on benefits of implementation science and to support sustainability and scalability of interventions and implementation strategies.

This project has a major focus on increasing health equity through evaluation of implementation and patient outcomes for Black patients, rural residents, and/or patients living in poverty. The inclusion of these subset analyses will help to uncover potential disparities that may naturally occur during implementation. This is particularly important for remote symptom monitoring given the reliance on technology, which may not be readily accessible to all patients. While the project leverages philanthropic funding for telephones and navigator support to overcome such barriers at the onset, additional strategies will likely be needed to support dissemination of this intervention to diverse populations. Findings on implementation strategies to support these patients may be applicable to future technology-supported applications.

A limitation of this study is the potential for missing data. EMR data often have missing variables and claims data will not be available for all patients. However, the benefits of the pragmatic evaluation are anticipated to outweigh these limitations in this study for several key reasons. First, this approach allows for inclusion of all patients in the participating institutions, which would not be feasible from a logistical perspective if primary data collection were required. Second, the project provides a measurement strategy with delineation of real-world data sources that could be abstracted by any practice without excessive cost. This provides substantial value for future practices who will track their own implementation and effectiveness of implementation strategies as part of local quality improvement efforts. Another potential limitation is the reliance on non-study personnel who may have high turnover or be tasked with other duties, particularly in light of the ongoing pandemic. We will monitor and document these real-world challenges, identifying implementation strategies to address them where appropriate, which will be disseminated through the implementation blueprint. Future studies will consider how implementation strategies differ across a national cohort of sites implementing remote symptom monitoring as part of standard of care.

## Supplementary Information

**Additional file 1.** Provider Semi-structured Interview Guide – This file contains the interview guide that will used for each of the Provider interviews that will be conducted.

**Additional file 2.** ePRO Patient Interview Guide – This file contains the interview guide that will be used for each of the patient interviews that will be conducted.

**Additional file 3.** UAB IRB Stamped Consent Form for the Patient Interview – This file contains the IRB approved consent form that patients will review and sign if they agree to participate in the patient interview.

## Data Availability

No data are available for the protocol, data from project are anticipated to be made available upon request.

## Abbreviations

BCBS-AL: Blue Cross Blue Shield of Alabama
CFIR: Consolidated Framework for Implementation Research
ED: Emergency Department
EMR: Electronic Medical Record
ePRO: Electronic Patient-Reported Outcomes
ERIC: Expert Recommendations for Implementing Change
FRAME: Framework for Modifications and Adaptions
MCI: University of South Alabama Mitchell Cancer Institute
OCM: Oncology Care Model
PRO-CTCAE: ePRO version of the Common Terminology Criteria for Adverse Events
PROmpt: Remote Symptom Monitoring Platform.
RUCA: Rural-Urban Commuting Area
UAB: University of Alabama at Birmingham
