# Supplementary material for: Evaluating the implementation and impact of navigator-supported remote symptom monitoring and management: a protocol for a hybrid type 2 clinical trial
Source: BMC Health Serv Res. 2022 Apr 22;22:538. doi: 10.1186/s12913-022-07914-6 (PMC9027833; doi:10.1186/s12913-022-07914-6)
Supplement: Supplementary file 1 — Additional file 1. Provider Semi-structured Interview Guide – This file contains the interview guide that will used for each of the Provider interviews that will be conducted. [file 12913_2022_7914_MOESM1_ESM.docx]

**Clinician Semi-Structured Interview guide**

Good [morning/afternoon/evening.]. My name is [TBN] and I will be conducting today’s interview. You have been invited to participate in this study because you provide care for patients with cancer. We are working to better understand the implementation of patient-reported outcomes as a part of standard of care.

For today’s discussion, we are interested in hearing about your perspectives on the implementation of our home symptom monitoring program, in which patients complete weekly surveys and the care team manages alerts from the surveys. This is also sometimes called ePROs, Carevive, or PROMPT. Please know that there is no right or wrong answer here today. We want to hear your ideas on this and learn about your experiences. Please be open and candid with us.

Before we start, I want to tell you that everything you say during your conversation with me today will remain confidential. Your responses will not be shared with anyone outside the research team. We may use data, without your name or other identifiable information, in quality improvement reports and publications. Do I have your permission to record our conversation, so I can more fully capture your responses to these questions?

Do you have any questions before we begin?

### Interview Questions

1. What is your current role taking care of cancer patients?
2. Can you tell me about how symptoms are managed at [institution]?
   1. When patients call?
   2. What about in-person clinic?
   3. Utilizing nurses/care team members?

3. Can you tell me how this has been rolled out at your institution? a. how would you describe this to someone who has never heard of it?

1. What has been your experience with ePROS/home-based symptom monitoring to date?
   1. What is your role?
   2. What do you like about our current strategy of home-based symptom monitoring?
   3. What would you like to see changed or improved?
2. What do you see as the challenges for you to complete your part in home-based symptom monitoring?

a. time commitment?

b. training on ePROS?

1. What helps you be able to complete your part of the home-based symptom monitoring?
2. Has the team reorganized their work for ePROs/home-based symptom monitoring?
   1. If not, why not?
3. What’s your perspective on the team’s understanding of their roles and responsibilities in ePROs/home-based symptom monitoring?
   1. Did ways of working as a team need to change?
4. What, if any, additional resources were required for this program?
   1. If not, what resources that already existed were used to help?
5. We have used a number of strategies to make the ePROs/home-based symptom monitoring easier. What aspects of the roll out process have you found to be useful or effective?
   1. Next, I would like to go through several strategies we have used during the roll out. Would you recommend we keep using the following rollout strategies? Why or why not?
      1. Messaging and organization from leadership on the program?
      2. Were there any champions for the program? Physician? Nurse?
         1. What role did/would the champion play?
         2. How did/would they work with you?
         3. What makes them a good champion?
      3. Standardizing this across all cancer types?
      4. Rolling out by disease group?
      5. Breaking up of tasks so that one team member doesn’t have to do everything (e.g. coordinator signs up patient, nurse responds to symptoms)
      6. Scripting (navigators)? Videos? Pamphlets?
      7. Integration to electronic medical record? Automated notes to reduce documentation?
      8. Changes to reduce alert fatigue (nurses) – use of “snooze” feature, outpatient only management of symptoms, removing fatigue as symptom, stopping surveys when patient leaves
      9. Availability of phones for patients with poor access?
      10. Monthly monitoring reports fed back to the clinic?
      11. Are there other strategies that you think would make this process easier for you?

11. How was the training process for you?

a. What were the most critical elements to provide training on? b. Was it clear from training which team member had which responsibility in the program?

c. Hands-on training in clinic vs. via Zoom?

12. What do you think will help sustain use of the ePROs/home-based symptom monitoring for our patients?

a. Anything else?

13. How has the ePROs/home-based symptom monitoring program helped you improve symptom management for patients?

a. Have you used the dashboard of patient’s symptoms to discuss these symptoms with your patients?

b. Have you incorporated the symptom monitoring care plan in your practice?

c. How has home symptom monitoring affected your patients’ prognosis or quality of life? If not, why not?

14. What changes have you or others made to adapt to COVID-19?

15. Are there other ways we could make this process easier for our patients?

a. Nurses? Doctors? Trainees?

16. Are there any other concerns/recommendations that we have not talked about that you would like to share?

Thank you very much for taking the time to participate in this study.
