# Supplementary material for: Evaluating the implementation and impact of navigator-supported remote symptom monitoring and management: a protocol for a hybrid type 2 clinical trial
Source: BMC Health Serv Res. 2022 Apr 22;22:538. doi: 10.1186/s12913-022-07914-6 (PMC9027833; doi:10.1186/s12913-022-07914-6)
Supplement: Supplementary file 2 — Additional file 2. ePRO Patient Interview Guide – This file contains the interview guide that will be used for each of the patient interviews that will be conducted. [file 12913_2022_7914_MOESM2_ESM.docx]

**Patient Semi-Structured Interview guide**

Good [morning/afternoon/evening.]. My name is [TBN] and I will be conducting today’s interview. You have been invited to participate in this study because you are a patient with cancer.

Thank you for participating in this research study. As we discussed, this interview will be recorded so that we can review your responses after this interview. Are you okay with this interview being recorded?

The purpose of this study is to understand your experiences of the PROmpt Home based Symptom Monitoring (ePRO) Program. This program involves completing weekly symptom surveys on your phone or your computer. We are looking for your input for what your health care team can do to help provide timely symptom management for your cancer care. This interview will last about 30 minutes. I will ask you a series of open-ended questions. Some of the questions may sound a little repetitive, simply because they are targeting different components of the program, and we really want to learn about your experience surrounding these different components. Please share as you feel comfortable. Your responses to these questions are completely confidential. I will try not to talk a lot and listen to you. If at any time you have a question, or would like to take a break, please let me know. Do you have any questions before we begin?

For today’s interview, we are interested in hearing what you think about this program, including what has made it helpful or challenging. Please know that there are no right or wrong answers and that everyone’s perspective is unique and important. We want to hear *your* ideas on this and learn about your experiences. Please be open and candid during the interview as this can help us improve the program.

Before we start, I want to tell you that everything you say during your conversation with me today will remain confidential. Your responses will not be shared with anyone outside the research team. We may use data, without your name or other identifiable information, in quality improvement reports and publications.

Do you have any questions before we begin?

(*If no questions or after questions are resolved):* Okay, if you have any questions as we go, please feel free to stop and ask me at any time.

### A. Interview Questions

1. Tell me a little bit about your cancer

a. how long ago

b. type of cancer

2. Since receiving care at [institution], how have symptoms affected your day to day life? How have you been managing them?

3. What has been your overall experience with the weekly symptom surveys to date?

Beginning vs now?

b. On a scale of 0 to 10, how would you rate your satisfaction of using the weekly symptom monitoring, 0 being the lowest satisfaction, 10 being the highest. c. How have they been helpful? What about them could be better?

4. How did you become enrolled in the weekly symptom monitoring? Can you tell me about how (your care guide/navigator/coordinator or use name) signed you up?

- 1. What made this process easier/harder? (explained thoroughly, quick process, video, use of a person to assist, doing the survey right away at sign-up)
  2. Was there anything that you didn’t like about this process? (unclear, took too long)
  3. Was the person who signed you up the right member of the care team to do this task? For example, this role could have been served by the front desk or the nurse.
  4. Is there anything else that you recommend that we could do to make this easier?

5. What has it been like filling out the weekly symptom surveys at home?

a. What made this easier/harder (automated reminders, option for text/email, log-in problems, seemed relevant to their care)?

b. Was there anything that you didn’t like about this process?

c. Is there anything we could improve or that you recommend?

6. What helped you remember to complete the survey?

a. If yes, can you tell me about this process? b. What made this easier/harder? c. Was there anything that you didn’t like about this process? d. Is there anything we could improve or that you recommend?

7. How did your care team use what you report on your weekly symptom surveys? Did someone on your care team communicate with to you about the symptoms you reported?

a. Was the discussion in clinic/over phone/portal message?

b. Which members of the care team did you discuss the reported symptoms with? c. Was there anything that you didn’t like about this process?

d. Is there anything we could improve?

e. Your physician has a dashboard of your symptoms- have they used this to discuss your symptoms with you?

8. In the platform, there is a symptom management care plan available for you. Have you looked at this care plan?

a. How was your experience using the symptom monitoring plan generated on the website based on your symptoms?

If Yes

a. What did you like about this? How did you use this?

b. Was there anything that you didn’t like?

c. Is there anything we could improve or that you recommend?

If No

a. Can you tell me why you did not access the plan? (Referral to care team if needed)

b. Did you have issues accessing the plan?

9. We have used a number of approaches to help roll out these weekly symptom surveys. I’d like to name each one and hear what you thought of each of them. What is your experience with [INSERT APPROACH]?

a. The introduction video that tells you about the Home Symptom Monitoring Program and the sign-up information b. Having your (navigator/careguide/care coordinator) help you with the sign up c. The weekly reminder texts d. Having your (navigator/careguide/care coordinator) call/text you a reminder to complete weekly surveys e. Materials (magnet, pamphlet to help remember to take surveys)

10. What else helped you participate in our weekly symptom surveys?

For patients who declined or dropped out:

1. Can you tell me a little about why you decided not to participate in the weekly symptom surveys?
2. What was difficult about this process?
3. How could we have made this easier for you?
4. Do you have any other suggestions for us to help patients participate?
5. Some participants we’ve talked to so far have talked about… What has been your experience?
6. Is there anything else you would like to add before we go?

Thank you very much for taking the time to participate in this study.
