# Supplementary material for: Evaluating the implementation and impact of navigator-supported remote symptom monitoring and management: a protocol for a hybrid type 2 clinical trial
Source: BMC Health Serv Res. 2022 Apr 22;22:538. doi: 10.1186/s12913-022-07914-6 (PMC9027833; doi:10.1186/s12913-022-07914-6)
Supplement: Supplementary file 3 — Additional file 3. UAB IRB Stamped Consent Form for the Patient Interview – This file contains the IRB approved consent form that patients will review and sign if they agree to participate in the patient interview. [file 12913_2022_7914_MOESM3_ESM.pdf]

CONSENT FORM TO BE PART OF A RESEARCH STUDY

Title of Research: **Evaluating the implementation and impact of navigator-delivered ePRO home symptom monitoring and management**

UAB IRB Protocol #: IRB-300007406

Principal Investigator: Gabrielle B. Rocque, MD, MSPH

Sponsor: NIH - National Institutes of Health/DHHS

**General Information**	You are being asked to take part in a research study. This research study is voluntary, meaning you do not have to take part in it. The procedures, risks, and benefits are fully described further in the consent form.
**Purpose**	Purpose is to evaluate the implementation and outcomes associated with standard-of-care Home ePRO
**Duration & Visits**	You will be in this study for 30 minutes – 1 hour
**Overview of Procedures**	Complete a demographic form and participate in a 30 minutes – 1 hour long interview.
**Risks**	The greatest risk is loss of confidentiality.
**Benefits**	You will not benefit directly from taking part in this study. However, potential benefits from participation in this project include increased understanding of Home ePRO effectiveness.
**Alternatives**	If you do not want to take part in the study, then your alternative is not to participate.

Purpose of the Research Study

We are asking you to take part in a research study. The purpose is to evaluate the implementation and outcomes associated with standard-of-care Home electronic Patient Reported Outcomes (ePRO). This study will include patients, who bring a broad perspective to how the patient experience should be considered in research. We are planning to enroll approximately 15-60 participants from the University of Alabama at Birmingham (UAB) and University of South Alabama (USA) Mitchell Cancer Institute (MCI).

Study Participation & Procedures

If you participate the study, you will be asked to fill out a demographic form and participate in an interview. The demographic form will include questions about your age, sex, race and education. The demographic form will take 5 minutes to complete. During the interview, you will be asked questions to describe your perspective on the patients completing patient-reported outcomes, including acceptability, and barriers to implementation of home ePROs. This interview will take place in the clinic room or in a separate private room within the UAB O'Neal Comprehensive Cancer Center, and will take 30 minutes to 1 hour of your time. Your interview session will be audio-recorded on secure and encrypted devices.

Risks and Discomforts

The greatest risk is breach of confidentiality. The study staff will protect your records so that your name, address, phone number will be kept private and will not release this information to anyone outside of

authorized study personnel. The chance that this information will be given to someone outside of the authorized study personnel is very small.   Although it may be uncomfortable talking and answering some questions about your cancer experience. You do not have to answer any questions that you do not wish to answer.

Benefits
You will not benefit directly. However, potential benefits from participation in this project include project include increased understanding of Home ePRO effectiveness. Knowledge gained from this study may help improve these activities for future cancer patients.

Alternatives
Your alternative is not to participate in this study.

Confidentiality and Authorization to Use and Disclose Information for Research Purposes
Federal regulations give you certain rights related to your health information. These include the right to know who will be able to get the information and why they may be able to get it. The study doctor must get your authorization (permission) to use or give out any health information that might identify you.

What protected health information may be used and/or given to others?
All medical information, including but not limited to information and/or records of any diagnosis or treatment of disease or condition, which may include sexually transmitted diseases (e.g., HIV, etc.) or communicable diseases, drug/alcohol dependency, etc.; all personal identifiers, including but not limited to your name, social security number, medical record number, date of birth, dates of service, etc.; any past, present, and future history, examinations, laboratory results, imaging studies and reports and treatments of any kind, including but not limited to drug/alcohol treatment, psychiatric/psychological treatment; financial/billing information, including but not limited to copies of your medical bills; any other information related to or collected for use in the research study, regardless of whether the information was collected for research or non-research (e.g., treatment) purposes; records about any study drug you received or about study devices used; and consent forms from past studies that might be in your medical record.

A description of this clinical trial will be available on www.ClinicalTrials.gov, as required by U.S. Law. This website will not include information that can identify you. At most, the website will include a summary of the results. You can search this website at any time.

Who may use and give out information about you?
Information about your health may be used and given to others by the study doctor and staff. They might see the research information during and after the study.

Who might get this information?
All Individuals/entities listed in the informed consent document(s), including but not limited to, the physicians, nurses and staff and others performing services related to the research (whether at UAB or elsewhere). Your information may also be given to the sponsor of this research. “Sponsor” includes any persons or companies that are working for or with the sponsor, or are owned by the sponsor, or are providing support to the sponsor (e.g., contract research organization).

Information about you and your health which might identify you may be given to:
- the Office for Human Research Protections (OHRP)

- the U.S. Food and Drug Administration (FDA)
- Department of Health and Human Services (DHHS) agencies
- Governmental agencies in other countries
- Governmental agencies to whom certain diseases (reportable diseases) must be reported
- the University of Alabama at Birmingham - the physicians, nurses and staff working on the research study (whether at UAB or elsewhere); other operating units of UAB, UAB Hospital, UAB Highlands Hospital, *University of Alabama Health Services Foundation,* , as necessary for their operations; the UAB IRB and its staff
- the billing offices of *UAB and UAB Health Systems affiliates* and its billing agents

Why will this information be used and/or given to others?
Information about you and your health that might identify you may be given to others to carry out the research study. The sponsor will analyze and evaluate the results of the study. In addition, people from the sponsor and its consultants will be visiting the research site. They will follow how the study is done, and they will be reviewing your information for this purpose.

This research is covered by a Certificate of Confidentiality from the National Institutes of Health. The researchers with this Certificate may not disclose or use information, documents, or biospecimens that may identify you in any federal, state, or local civil, criminal, administrative, legislative, or other action, suit, or proceeding, or be used as evidence, for example, if there is a court subpoena, unless you have consented for this use. Information, documents, or biospecimens protected by this Certificate cannot be disclosed to anyone else who is not connected with the research except, if there is a federal, state, or local law that requires disclosure (such as to report child abuse or communicable diseases but not for federal, state, or local civil, criminal, administrative, legislative, or other proceedings, see below); if you have consented to the disclosure, including for your medical treatment; or if it is used for other scientific research, as allowed by federal regulations protecting research subjects.

The Certificate cannot be used to refuse a request for information from personnel of the United States federal or state government agency sponsoring the project that is needed for auditing or program evaluation by the NIH - National Institutes of Health/DHHS which is funding this project or for information that must be disclosed in order to meet the requirements of the federal Food and Drug Administration (FDA). You should understand that a Certificate of Confidentiality does not prevent you from voluntarily releasing information about yourself or your involvement in this research. If you want your research information released to an insurer, medical care provider, or any other person not connected with the research, you must provide consent to allow the researchers to release it.

The Certificate of Confidentiality will not be used to prevent disclosure as required by federal, state, or local law.

The Certificate of Confidentiality will not be used to prevent disclosure for any purpose you have consented to in this informed consent document.

What if I decide not to give permission to use and give out my health information?
By signing this consent form, you are giving permission to use and give out the health information listed above for the purposes described above. If you refuse to give permission, you will not be able to be in this research.

May I review or copy the information obtained from me or created about me?
You have the right to review and copy your health information. However, if you decide to be in this study and sign this permission form, you will not be allowed to look at or copy your information until after the research is completed.

May I withdraw or revoke (cancel) my permission?
Yes, but this permission will not stop automatically. The use of your personal health information will continue until you cancel your permission.

You may withdraw or take away your permission to use and disclose your health information at any time. You do this by sending written notice to the study doctor. If you withdraw your permission, you will not be able to continue being in this study.

When you withdraw your permission, no new health information which might identify you will be gathered after that date. Information that has already been gathered may still be used and given to others. This would be done if it were necessary for the research to be reliable.

Is my health information protected after it has been given to others?
If you give permission to give your identifiable health information to a person or business, the information may no longer be protected. There is a risk that your information will be released to others. Including others outside of UAB, without your permission.

Voluntary Participation and Withdrawal
Whether or not you take part in this study is your choice. There will be no penalty if you decide not to be in it. If you decide not to be in the study, you will not lose any benefits you are otherwise owed.

You are free to withdraw from this study at any time. Your choice to leave the study will not affect your relationship with this institution. Contact the study doctor if you want to withdraw from the study.

If you are a UAB student or employee, taking part in this research is not a part of your UAB class work or duties. You can refuse to enroll, or withdraw after enrolling at any time before the study is over, with no effect on your class standing, grades, or job at UAB. You will not be offered or receive any special consideration if you take part in this research.

Cost of Participation
There will be no cost to you for taking part in this study.

Payment for Participation
You will receive $50 for your participation in the study. Ask the study staff about the method of payment that will be used for this study (e.g., check, cash, gift card, direct deposit).

Questions
If you have any questions, concerns, or complaints about the research, you may contact Dr. Gabrielle Rocque at by telephone at (205) 996-9281 or the study manager Stacey Ingram by telephone at (205) 934-5287. They will be glad to answer any of your questions.

If you have questions about your rights as a research participant at UAB, or concerns or complaints about the research, you may contact the UAB Office of the IRB (OIRB) at (205) 934-3789 or toll free at 1-855-860-3789. Regular hours for the OIRB are 8:00 a.m. to 5:00 p.m. CT, Monday through Friday.

Legal Rights
You are not waiving any of your legal rights by signing this consent form.

Signatures
Your signature below indicates that you have read (or been read) the information provided above and agree to participate in this study. You will receive a copy of this signed consent form.

____

Signature of Participant **Date**

____

Signature of Person Obtaining Consent **Date**
